# Pharmacological Modulation of Behaviour, Serotonin and Dopamine Levels in *Daphnia magna* Exposed to the Monoamine Oxidase Inhibitor Deprenyl

**DOI:** 10.3390/toxics9080187

**Published:** 2021-08-09

**Authors:** Marina Bellot, Melissa Faria, Cristian Gómez-Canela, Demetrio Raldúa, Carlos Barata

**Affiliations:** 1 Department of Analytical Chemistry and Applied (Chromatography Section), School of Engineering, Institut Químic de Sarrià-Universitat Ramon Llull, Via Augusta 390, 08017 Barcelona, Spain; marina.bellot@iqs.url.edu (M.B.); cristian.gomez@iqs.url.edu (C.G.-C.); 2 Institute for Environmental Assessment and Water Research (IDAEA-CSIC), Jordi Girona 18, 08034 Barcelona, Spain; mdfqam@cid.csic.es (M.F.); drpqam@cid.csic.es (D.R.)

**Keywords:** *Daphnia magna*, neurotransmitter, modulation, pharmaceuticals

## Abstract

This study assessed the effects of the monoamine oxidase (MAO) inhibitor deprenyl in *Daphnia magna* locomotor activity. The mechanisms of action of deprenyl were also determined by studying the relationship between behaviour, MAO activity and neurotransmitter levels. Modulation of the *D. magna* monoamine system was accomplished by 24 h exposure to two model psychotropic pharmaceuticals with antagonistic and agonistic serotonin signalling properties: 10 mg/L of 4-chloro-DL-phenylalanine (PCPA) and 1 mg/L of deprenyl, respectively. Contrasting behavioural outcomes were observed for deprenyl and PCPA reflected in decreased basal locomotor activity and enhanced habituation for the former compound and delayed habituation for the latter one. Deprenyl exposure inhibited monoamine oxidase (MAO) activity and increased the concentrations of serotonin, dopamine and the dopamine metabolite 3-methoxytyramine in whole *D. magna* extracts. Our findings indicate that *D. magna* is a sensitive and useful nonvertebrate model for assessing the effects of short-term exposure to chemicals that alter monoamine signalling changes.

## 1. Introduction

Animal behaviour to environmental stimuli changes such as predation or food availability is a key fitness trait [1]. Of particular interest are behavioural responses related to predator avoidance such as sudden locomotion changes in response to visual or tactile stimuli [2,3]. Any changes in the normal behavioural conduct could compromise individual survival. Neurotransmitters modulate behavioural plasticity at the molecular level. Serotonin is one of the major neurotransmitters in the central nervous system (CNS), modulating many behaviours including perception, mood, reward, anger, aggression, appetite, memory, sexuality and attention [4]. Neurological pathologies such as schizophrenia, depression and anxiety have been related to dysfunctions in the serotonergic system. The serotonergic system is well-known in vertebrates, but little is known about many invertebrates. Tryptophan hydroxylase (TPH) is the enzyme that converts tryptophan to 5-hydroxytryptophan (5-HTP), which is the rate-limiting step of serotonin synthesis. When serotonin is released to the synaptic cleft, the serotonin transporter SERT mediates its uptake/reuptake to the serotonergic neurons, where monoamine oxidase (MAO) metabolizes serotonin to 5-hydroxy-indolecetaldehyde, which is quickly metabolized by aldehyde dehydrogenase to form 5-hydroxyindoleacetic acid (5-HIAA), the major metabolite of serotonin [5].

Many of the emerging contaminants present in surface waters are neuroactive substances required for highly prescribed drugs to control cardiac and neurological disorders [6]. Neuroactive compounds targeting the serotonergic system are of particular concern due to the essential role of serotonin in both neurotransmission and neuromodulation. Thus, it is not surprising to find many investigations on the effects of selective serotonin reuptake inhibitors (SSRIs) in nontarget aquatic species like fish but also in invertebrates [7,8]. Nevertheless, information regarding other potential modes of actions, such as TPH or monoamine oxidase (MAO) inhibition, is still scarce and almost absent for many invertebrates. In this regard, the ecotoxicological model species *Daphnia magna* is a good candidate for studying the role of serotonergic signalling pathways in behaviour and its modulation by neuroactive pollutants. *D. magna* is probably the most widely use organism in aquatic toxicological evaluations. It is a well-known ecological model and has its genome fully sequenced and annotated [9]. Therefore, *D. magna* offers the opportunity to study molecular and apical effects of pharmaceuticals. Furthermore, about 80% of the molecular human drug targets are also present in the *Daphnia* genome [10]. This means that it is likely that many neuroactive pollutants affect this organism. Recently, it was shown that CRISPR/Cas9-mediated tryptophan hydroxylase (TPH) knockout clonal *D. magna* lines lack serotonin and show an abnormally high basal swimming activity and a markedly reduced habituation to repetitive light stimuli [2,11]. The selective serotonin reuptake inhibitor (SSRI) fluoxetine that is the active ingredient of Prozac increases the serotonin levels in the brain of *D. magna* and increases reproduction [12]. Conversely, chloro-DL-phenylalanine (PCPA), which is an inhibitor of TPH, decreases serotonin levels in *D. magna* [13]. Fluoxetine and PCPA, however, despite having opposite effects on serotonin levels, have similar effects on *Daphnia* cognitive behaviour, decreasing habituation to repetitive light stimuli [2]. The aim of this study was to better characterize the serotonergic system in *Daphnia*. In particular, we studied the mechanisms of action of monoamine oxidase (MAO) inhibitors, such as deprenyl, in *D. magna*, addressing its effects on the target MAO enzyme activity, neurotransmitter levels and behavioural responses. Our hypothesis is that deprenyl should increase serotonin and probably also dopamine levels in *D. magna* and have behavioural responses different from those of the drugs that depress serotonin levels, such as PCPA. 

## 2. Materials and Methods

### 2.1. Experimental Animals 

Five-day old juveniles from a single clone of *D. magna* (clone F) were used for exposure, behavioural and biochemical determinations assays. Further details of culture conditions are provided in the Supplementary Materials, Section S1.1.

### 2.2. Experimental Procedures

Deprenyl (CAS:14611-52-0) and chloro-DL-phenylalanine (PCPA; CAS: 7424-00-2) were of high-quality grade and purchased from Sigma-Aldrich (St. Louis, MO, USA). Stock solutions were prepared in the Milli-Q water and then diluted in ASTM hard water. *D. magna* juveniles (5-days-old) were pre-exposed to the selected compounds in ASTM hard water without food for 24 h. The selected chemicals concentrations were far below those having detrimental effects on survival or swimming (>20 mg/L; [2]). The compounds were initially screened for light stimuli motile responses using a broad concentration range ranking from 0.1 to 1000 µg/L for deprenyl and from 0.1 to 10,000 µg/L for PCPA [2]. The concentrations having the greatest effect (1 and 10 mg/L for deprenyl and PCPA, respectively) were used in the subsequent light stimuli motile response assays. For the behavioural assessment, exposures were conducted in groups of 12 individuals in 300 mL of the test medium in 500 mL glass vessels. Following 24 h of exposure, 12 animals were distributed randomly to 24-well plates (two treatments per plate). From 12 to 24 individual replicates were performed for each tested chemical. For the MAO activity and neurotransmitters assessment, exposures were carried out in groups of 20 or 5 individuals in 500 mL or 100 mL of the medium, respectively. The treatments were replicated five times. Following exposure, the individuals from each replicate (20 or 5) were pooled in an Eppendorf, the water was removed and the rest was deep-frozen in liquid N_2_. The samples were stored at −80 °C until analysis. For each behavioural assay and MAO determination, juveniles were collected from 2–4 trials of the same experimental setup conducted on different days and with different batches of animals.

### 2.3. Behavioural Analysis

The *Daphnia* photomotor response assay (DPRA) was performed as described in [2]. Details of the assay are provided in the Supplementary Materials, Methods, Section S1.2. The assay measured the distance moved after a sudden increase in light intensity across 30 repetitive light stimuli of 1 s followed by 4 s of darkness. Following a previous study, “enhanced photomotor response” (EPR) is defined as the area under the curve (AUC_EPR_) for the first 10 stimuli where the response to light increases. Conversely, “habituation or non-associative learning” is defined as the area under the curve (AUCh) for the decreasing responses to stimuli [2]. 

To better characterize the swimming activity under darkness and upon continuous light, basal locomotor activity (BLM) and visual motor response (VMR) analyses of 5-days-old *D. magna* juveniles were also assessed using the same DanioVision system device as described below. Before the video recording, the juveniles were first acclimated for 10 min under dark conditions. Video tracking trials consisted of a 10 min cycle with a 5 min dark period followed by a 5 min light period (290 lux). The basal locomotor activity (BLM) was defined as the total distance (mm) travelled by the juveniles during the last minute of the first dark cycle. The visual motor response (VMR) was based in the hyperactivity period induced by light, which in *D. magna* increased during the first minutes and decreased afterwards. 

### 2.4. Daphnia Monoamine Oxidase (MAO) Activity

*D. magna* juveniles were collected in pools of 20 individuals and homogenized in ice-cold 10 mM phosphate buffer (pH 7.6) supplemented with 1 mM EDTA using a TissueLyser^®^ (Qiagen, Germantown, MA, USA). The volume of the buffer was adjusted to 100 juveniles/mL. Following centrifugation at 2500× *g*, 4 °C for 5 min, MAO activity was determined in the supernatant according to Faria et al. [14]. Further information is provided in the Supplementary Materials, Section S1.3.

### 2.5. Extraction and Analysis of Neurotransmitters

Monoaminergic neurotransmitters were extracted from pools of five juveniles according to the procedure adapted from the article by Fuertes et al. [13]. Additional details on the extraction and analysis of neurotransmitters are provided in the Supplementary Materials, Section S1.4.

### 2.6. Statistical Analysis

The experimental design for behavioural assay responses of controls were compared to those of treatments using either the Student’s *t*-test when only controls and deprenyl treatments were used or one-way ANOVA followed by Dunnett’s post-hoc test when PCPA was also considered. MAO activities and concentration of metabolites of the unexposed juveniles and the deprenyl-treated ones were compared using the Student’s *t*-test. For the *Daphnia* photomotor responses, the area under the curve AUC_EPR_ and AUC_h_ values obtained for the individual juveniles across the repetitive light stimuli were used for statistical comparisons. The basal and visual locomotor activities of the *D. magna* juveniles monitored during 5 min of dark and 5 min of light were analysed simultaneously using repeated measures ANOVA considering the total distance moved in the last minute of darkness and in each of the five minutes of the light period as the repeated measures (hereafter referred to as the “time”). Prior to the analyses, ANOVA assumptions of data normality and variance homoscedasticity were tested. Analysis of the data was performed with IBM SPSS v25 (Statistical Package 2010, Chicago, IL, USA). Significance was set at *p* < 0.05.

## 3. Results

### 3.1. Behaviour

**Figure 1:**
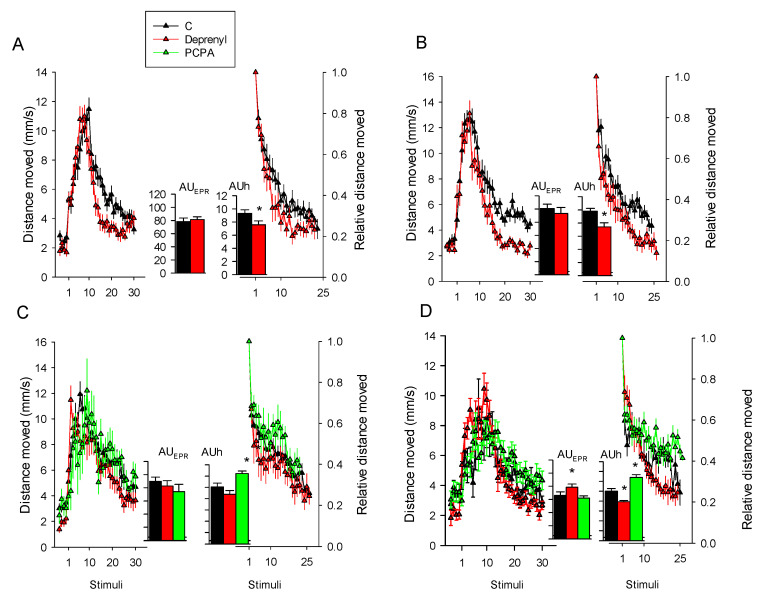
*Daphnia* photomotor responses to repetitive light stimuli following 24 h exposures to 1 and 10 mg/L of deprenyl and PCPA, respectively. Plots of the average distance moved ± SE (*n* = 12–24) against 30 tapping stimuli at 5 s ISI and corresponding bar charts (graph inlet) of the calculated area under the curve (mean ± SE) for EPR (AUC_EPR_) and habituation AUCh phases. Within each of the four graphs, the left plots represent the full motile responses whereas the right ones show habituation responses measuring the decrease in the distance moved (proportions) relative to the maximum response to the light stimulus delivered (set to 1). Graphs (**A**,**B**) depict the data obtained across two independent experiments for deprenyl exposures, (**C**,**D**)—for deprenyl and PCPA exposures. Within the AUC bar graphs, * means significant (*p* < 0.05) treatment differeces relative to the controls following the Student’s *t*-test or ANOVA and Dunnett’s test. Axis scales for the AUC (graph inlet) are depicted in graph (**A**).

For the *Daphnia* photomotor response assay (DPRA) to repetitive light stimuli, absolute and proportional distances moved across the tested compounds are reported in Figure 1 and the statistics are referred to the AUC values reported in the graph inlet. The statistics are depicted in Table 1. Deprenyl increased habituation significantly (*p* < 0.05) in three out of the four experiments (Figure 1A,B,D) and enhanced photomotor responses in only one of them (Figure 1D). PCPA reduced habituation in the two experiments that were performed and did not enhance photomotor responses (Figure 1C,D). 

**Figure 2:**
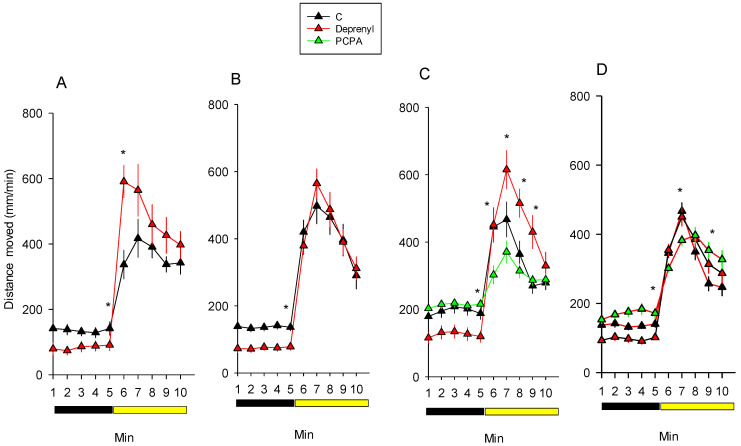
*Daphnia* basal locomotor activity (BLM) and visual motor responses (VMR) across 5 min in darkness and 5 min with light following 24 h exposures to 1 and 10 mg/L of deprenyl and PCPA, respectively. Plots of the average distance moved ± SE (*n* = 12–24) per minute across the 10 min of the monitored period are shown. Graphs (**A**,**B**) depict the data obtained across two independent experiments for deprenyl exposures and (**C**,**D**) for deprenyl and PCPA ones. Note: * during the minutes 5–10, mean significant (*p* < 0.05) treatment differences relative to the controls following the Student’s *t*-test or ANOVA and Dunnett’s test.

Basal locomotor activity (BLM) and visual motor response (VMR) analyses of 5-day-old *D. magna* juveniles are depicted in Figure 2. Repeated measures ANOVA denoted significant (*p* < 0.05) effects of treatment or of its interaction with time (Table 2). In all the experiments, deprenyl decreased the basal locomotor activity and in only half of them (Figure 2A,C) increased the response to light. PCPA decreased the response to light in the two experiments performed (Figure 2C,D) and increased the basal locomotor activity in one of them (Figure 2C).

### 3.2. Biochemical and Neurotransmitter Analysis 

**Figure 3:**
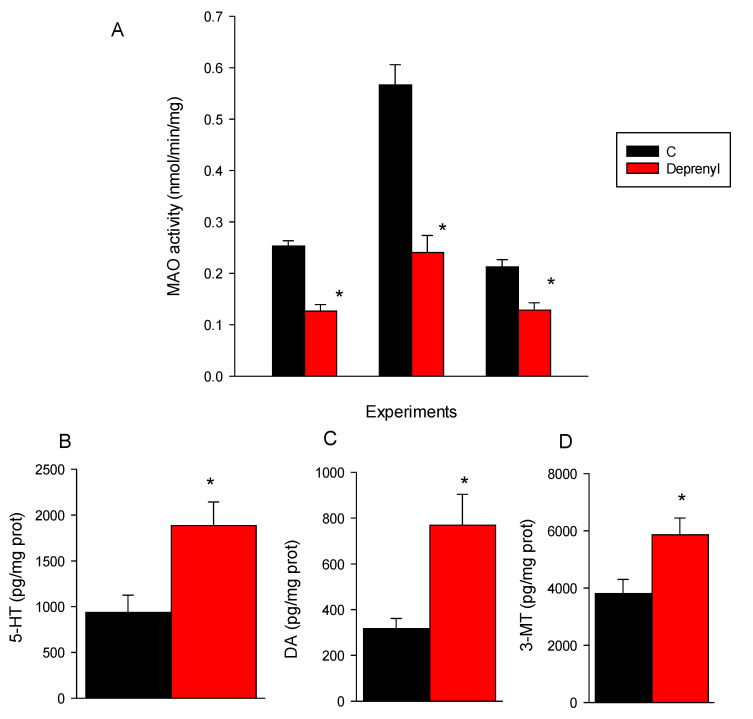
Effects over enzyme activity (**A**) and monoaminergic neurotransmitter levels (**B**–**D**) (means ± SE, *n* = 5–10) in the whole body of the *D. magna* juveniles following 24 exposures to 1 mg/L of deprenyl. For clarity, monoaminergic neurotransmitters that showed a significant change across treatments are depicted. The rest of the values are provided in Table S3, Supplementary Materials. Note: * the indicated bars mean significant (*p* < 0.05) treatment differences relative to the controls following the Student’s *t*-test.

MAO activity was significantly (*p* < 0.05) inhibited by deprenyl in the three experiments performed (Figure 3A), and deprenyl significantly (*p* < 0.05) increased serotonin (5-HT), dopamine (DA) and 3-methoxytyramine (3-MT) (Figure 3B–D). Details of the statistics and of nonsignificant metabolite values are provided in Tables S3 and S4, respectively, Supplementary Materials.

## 4. Discussion

In this study, we provided for the first time the results on the mode of action of deprenyl in *D. magna*. Deprenyl at 1 mg/L inhibited about 50% of the MAO activity and by doing so increased the concentrations of serotonin and dopamine by 2- and 2.4-fold, respectively. There are two types of MAO inhibitors, type A and type B. Type A inhibitors block the catabolism of noradrenaline and serotonin, and type B inhibitors block the catabolism of dopamine. Deprenyl is a MAO type B inhibitor in mammalian models; however, at high dosages, it inhibits both type A and type B MAO [15]. This means that deprenyl, apparently, has a similar mechanism of action as in vertebrates (i.e., mammals and fish) inhibiting MAO type B but also A activity and thus preventing the metabolism of serotonin and dopamine [14,16]. There is controversy on the role, if any, of the MAO types on the metabolism of monoamines in arthropods [17]. According to the previous review, alternative metabolic routes such as *N*-acetylation, γ-glutamyl conjugation, sugar conjugation, sulfation, β-alanyl conjugation are predominantly used by insects and crustaceans to metabolize monoamines. The reported evidence, however, indicates that ticks and mites have a unique MAO sensitive to deprenyl that catabolizes serotonin and dopamine [18,19]. Furthermore, in the hepatopancreas of the crab *Paralithodes camtschaticus*, there is also only one type of MAO that shows great inhibition specificity for deprenyl [20]. The *D. magna* genome contains a unique flavin-containing monoamine oxidase A gene/protein (ACC XP_032781527.1) that has 54% homology with a putative MAO protein of the shrimp *Penaeus vannamei* (XP_027230105.1), 33%—with human MAOs. These homologies seem moderate but note that the zebrafish MAO gene/protein has only 69% homology with those of humans. Thus, the results obtained in this study are in line with the previously reported studies on ticks, mites and crabs and provide evidence for the presence of a MAO-like activity in *D. magna* that is able to metabolize serotonin and dopamine.

Using similar concentrations of deprenyl (5 µM ≅ 0.94 mg/L), Faria et al. [14] reported similar background activities of MAO in zebrafish larvae, but an almost complete inhibition of MAO activity and a greater increase of serotonin than of dopamine. Phylogenetic differences between *D. magna*, fish and mammals are likely to account for the observed deprenyl specificity effect on the MAO activity and of the latter enzyme for catabolising serotonin and dopamine [21]. Deprenyl, despite decreasing the MAO activity and enhancing serotonin, did not decrease the serotonin degradation metabolite 5-hydroxyindoleacetic acid (5-HIAA). There are, however, reported discrepancies on the consequences of the MAO type A,B inhibition on serotonin degradation metabolites. The reported studies on the zebrafish exposed to deprenyl found enhanced or unchanged levels of 5-HIAA [14,22]. In rodents, MAO type A inhibition that lead to enhanced levels of serotonin did not necessarily affect its metabolite 5-HIAA [23].

Deprenyl increased, however, the concentration of the dopamine metabolite 3-methoxytyramine (3-MT) in *D. magna*, an effect previously reported for mice treated with the MAO type A inhibitor clorgyline [24].

In a previous study we found that neuroactive drugs do not always show the same target specificity in *D. magna* as in mammals [13]. In relation to this, we analysed up to 16 metabolites belonging to four neurological metabolic pathways (i.e., serotonin, catecholamine, cholinergic and GABAergic). The results obtained for deprenyl showed high specificity for its putative serotonergic and dopaminergic targets.

The study of behavioural responses indicates contrasting effects of deprenyl against compounds that are known that decrease serotonin in *Daphnia* such as PCPA [13]. Deprenyl increased habituation to repetitive light stimuli, which is a primary form of non-associative learning [25], and enhanced responses to light in 50% of the experiments performed (1 out of 4 in Figure 1; 3 out of 4 in Figure 2). Conversely, PCPA-treated organisms took longer to habituate to the repetitive light stimuli and responded to a lower extent to visual light stimuli. Interestingly, *D. magna* individuals exposed to deprenyl also had a lower basal activity, which was monitored under darkness. The behavioural features of PCPA are consistent with the reported higher basal activity and reduced habituation of CRISPR-mediated TPH-mutated *D. magna* juveniles that lack serotonin [11]. In zebrafish larvae exposed to deprenyl, Faria et al. [14] also reported a lower basal activity, a reduced response to light or tactile stimuli and increased habituation. In fish and also in mammals, increased serotonin levels have sedative-like anxiolytic effects [14,26], whereas in *D. magna*, it is unclear. The results obtained here in *D. magna* for deprenyl apparently agree with those of zebrafish [14] since in both species it decreases the basal locomotor activity and increases habituation. However, in fish, deprenyl also decreased the response to stimuli [14], which agrees with the reported decreased anxiety-like responses in rodents [26]. In *D. magna*, deprenyl either did not affect or enhanced the response to stimuli. In relation to this, it is important to remark that, unlike in zebrafish, deprenyl increased the dopamine level to the same extent as that of serotonin. Increased dopamine levels have been associated with a hyperresponsive behaviour to mechanical stimuli in *Drosophila melanogaster* and *Caenorhabditis elegans* [27]. Of course, dopamine does not act alone in regulating behavioural responsiveness to stimuli; there are counteracting neuronal systems, such as serotonin and other monoamines [27,28]. Therefore, the observed behavioural defects in the *D. magna* exposed to deprenyl are likely to be related to the observed enhanced levels of dopamine and serotonin.

In fish and also in mammals, decreasing serotonin levels induced by PCPA cause anxiety-like behaviour such as hyperlocomotion activity and enhanced responses to stimuli [14,29]. PCPA is also known to impair learning [30]. The *D. magna* exposed to PCPA showed basal hyperactivity only in one out of the two experiments, increased the response to light in some of the trials performed, but impaired learning in all the trials (decreased habituation). This means that the *D. magna* responses to PCPA can be related to anxiogenic behaviour only in part. Nevertheless, the previous results obtained with knockout *D. magna* lacking serotonin did show higher hyperactivity, enhanced responses to light stimuli and also impaired habituation [2,11], a phenotype compatible with anxiety-like behaviour and learning impairment in rodents [30,31]. The apparently closer phenotype of genetically modified *D. magna* individuals lacking serotonin than of those enzymatically impaired by PCPA can be related to the unspecific action of the latter compound. PCPA not only reduced serotonin in *D. magna* but also decreased the levels of norepinephrine [13], which is known to modulate arousal and other types of cognitive behaviour [32].

## 5. Conclusions

The results reported here show that *D. magna* juveniles are sensitive to MAO inhibitors that change serotonin signalling. In addition, molecular targets of MAO modulators such as effects on the enzymatic activity and on the concentration of serotonergic and dopaminergic metabolites was also observed. The model serotonin modulator deprenyl inhibited the MAO activity and increased the serotonin, dopamine and dopamine metabolite 3-MT levels. The deprenyl-treated individuals showed consistently shorter habituation to repetitive light stimuli and reduced basal locomotor activity. Deprenyl behavioural outcomes opposed those of the PCPA drug or genetically modified individuals having reduced serotonin levels. The findings presented in this study reinforce the use of this nonvertebrate model to address behavioural and physiological roles of serotonin.

## Figures and Tables

**Table 1:** Student’s *t*-test or one-way ANOVA results testing the effects of deprenyl and PCPA on the area under the curve (AUC) values obtained from *Daphnia* photomotor responses to repetitive light stimuli. AUCEPR and AUCh are, respectively, the areas of enhanced photomotor responses during the first 10 light stimuli and during habituation afterwards. Results for the four identical experiments are reported.

		df	t,F	*P*
Experiment 1	AUCh	31	2.1	0.047
	AUCEPR	31	0.4	0.657
Experiment 2	AUCh	31	3.3	0.003
	AUCEPR	31	0.7	0.493
Experiment 3	AUCh	2,45	10.2	<0.001
	AUCEPR	2,45	0.8	0.459
Experiment 4	AUCh	2,56	10.0	<0.001
	AUCEPR	2,56	3.9	0.026

**Table 2:** Results of repeated measures one-way ANOVA testing for the effects of treatment, monitoring time and its interaction (Time * Treatment) on the *D. magna* visual responses across the last minute of darkness and the 5 min of light. Results for four identical experiments are reported.

		df	F	*P*
Experiment 1	Time	1,22	16.6	0.001
	Treatment	1,22	4.4	0.048
	Time * Treatment	1,22	0.0	0.923
Experiment 2	Time	1,46	12.6	0.001
	Treatment	1,46	0.2	0.688
	Time * Treatment	1,46	4.2	0.047
Experiment 3	Time	1,69	10.9	0.002
	Treatment	2,69	8.7	<0.001
	Time * Treatment	2,69	5.7	0.005
Experiment 4	Time	1,68	17.9	<0.001
	Treatment	2,68	3.9	0.026
	Time * Treatment	2,68	4.3	0.017

## Data Availability

Data supporting the reported results will be provided upon reader’s request.

## Supplementary Materials

The following are available online at https://www.mdpi.com/article/10.3390/toxics9080187/s1, Supplementary Methods and Supplementary Results—Tables. Table S1: List the metabolites analysed, Table S2. Quality parameters of monoamine neurotransmitters and the related metabolites, Table S3: Student’s t-test results for MAO and monoaminergic neurotransmitter levels, Table S4: List of values for the nonsignificant metabolites.
