# Supplementary material for: Pharmacological Modulation of Behaviour, Serotonin and Dopamine Levels in Daphnia magna Exposed to the Monoamine Oxidase Inhibitor Deprenyl
Source: Toxics. 2021 Aug 9;9(8):187. doi: 10.3390/toxics9080187 (PMC8402476; doi:10.3390/toxics9080187)
Supplement: Supplementary file 1 [file toxics-09-00187-s001.zip › toxics-1321309-supplementary.pdf]

toxics

MDPI

Article

Supplementary Material: Pharmacological Modulation of Behaviour, Serotonin and Dopamine Levels in Daphnia magna exposed to the monoamine oxidase inhibitor deprenyl

Marina Bellot, Melissa Faria, Cristian Gómez-Canela, Demetrio Raldúa and Carlos Barata

1. Supplementary Methods

1.1. Experimental animals

Parthenogenetic cultures of a single clone of D magna (clone F) were used. This clone has a marked negative phototactic behaviour [1]. Photoperiod was set to 16 h light: 8 h dark cycle, and temperature at 20 ± 1 °C. Bulk cultures of 10 adult females were maintained in 2 L of ASTM hard synthetic water at high food ration levels (5x105 cells/mL of Chlorella vulgaris), following Barata and Baird [2]. Groups of 100 newborn individuals (< 24 h old) obtained from bulk cultures were reared in 2 L ASTM hard water plus algae for 5 days, and then used for exposure and behavioural assays. Cultures were renewed with new media every other day.

1.2. The Daphnia Photomotor Response Assay (DPRA)

The assays is based on measuring the distance moved after a sudden increase in light intensity [3]. By using DPRA it is possible to determine not only the magnitude of the phototactic response, but also its habituation after repetitive cycles of light and darkness. Video tracking was acquired, and EthoVision XT 9 software (Noldus, Wageningen, The Netherlands) was used to analyze the motile response. Trials were performed at 20 o C with near-infrared light. Light intensity of the stimuli was selected at 50% in DanioVision setting (290 lux) and, after a 10 min acclimation period to the chamber, 30 light stimulus of 1 s were delivered, one every 4 seconds. Videos were recorded at 30 frames per second and the Daphnia Photomotor Response (DPR) for each individual larva was analyzed by measuring the distance traveled (mm) over the 1 s period following each stimulus. Following a previous study "enhanced photomotor response" (EPR) is defined as the area under the curve (AUCEPR) for the first 10 stimulii and "Habituation or non-associative learning" as the area under the curve (AUCh) of plots of distance moved relative to the max response [2].

1.3. Daphnia magna monoamine-oxidase (MAO) activity

MAO activity was determined in the supernatant using the peroxidase-linked spectrophotometricassay described by Holt et al. [4]and adapted by Faria et al. [5], which quantify the amount of H2O2 released during amines oxidation. In this assay, 4-aminoantipyrine is oxidized and condensed with vanillic acid to produce a red quinoneimine dye. The assay was performed in a 96-well plate where 50 µL extracts were incubated in the presence of 100 µL of amine substrate tyramine, 10 mM final concentration and of 50 µL of a chromogenic solution containing final concentrations of 500 µM 4-aminoantipyrine, 1 mM vanillic acid and 4 U/mL horseradish peroxidase type II in 10 mM Phosphate Buffer pH 7.6. The reaction was left to stabilize for 15 min at room temperature and then incubated for a further 60 min at 28°C in the microplate reader (Synergy 2, Bio Tek) where the formation of the red quinoneimine dye was recorded at 490 nm. MAO activity results were presented as nmol/min/mg obtained using the molar absorption coefficient for quinoneimine dye at pH 7.6 (4656 M-1 cm-1) and normalized with total protein in assay.

1.4. Monoaminergic neurotransmitters extraction and analysis

1.4.1. Chemicals and materials

Crystalline pure solid standards of target metabolites were purchased from Sigma-Aldrich (St. Louis, MO, USA), Merck (Darmstadt, Germany), Toronto Research Chemicals (TRC, Toronto, Canada) and Tocris Bioscience (Ellisville, USA). All target metabolites are presented in Table S1. Furthermore, labelled standards such as L-tryptophan-1-13C, L-tyrosine-13C9, L-DOPA-2,5,6-d3 and DL-norepinephrine-d6 HCl were obtained from Toronto Research Chemicals (TRC, Toronto, Canada), whereas dopamine-1,1,2,2-d4 and acetylcholine-d9 were provided from Merck (Darmstadt, Germany) and 3-methoxytyramine-d4, phenylalanine-13C9,15N and choline-1-13C from Sigma-Aldrich (St. Louis, MO, USA).

Acetonitrile (ACN) and methanol (MeOH), both LC-MS grade, were supplied by VWR Chemicals Prolabo (Leuven, Belgium). However, formic acid (FA) was provided by Fischer Scientific (Loughborough, UK), while dimethyl sulfoxide (DMSO) and ammonium formate were supplied by Sigma-Aldrich (St. Louis, MO, USA). Ascorbic acid was obtained from Panreac AppliChem (Darmstadt, Germany). Finally, water was daily obtained through Millipore Milli-Q purification system from IQS facilities (Millipore, Bedford, MA, USA). Stock solutions of labeled and unlabeled compounds were prepared at 1 mg µL-1 in MeOH, DMSO or ultra-pure water depending on their solubility. Standard solutions of unlabeled metabolites were prepared at the desired concentration in starting mobile phase solvent, as well as the mixture of labelled standards (ISM), used as internal standard during the whole extraction process.

1.4.2. Extraction

Monoaminergic neurotransmitters were extracted from pools of 5 juveniles according to the procedure adapted from Fuertes et al. [6] based on the use of a solvent of similar polarity to that of the neurotransmitters in order to be extracted from the sample. Ultra-high-performance liquid chromatography (Acquity UPLCH-ClassWaters, Milford, MA, USA) coupled to a triple quadrupole mass spectrometer equipped with an electrospray (ESI) source (Xevo, TQS micro,Waters, Milford, MA, USA) was used to perform the analysis. This extraction procedure required working on ice or equivalent at low temperatures (between 0ºC and 4ºC) during all the process to prevent the possible degradation of target metabolites. Firstly, 500 µL of ACN + 0.1% FA + 0.02% ascorbic acid, 50 µL of ISM and three stainless steel beads were added in each polypropylene tubes (Eppendorf) containing the samples. Samples were shaken using a vortex mixer and were placed inside of a homogenizer (TissueLyser LT, Quiagen, Hilden, Germany) at 50 osc/min for 30 seconds. Then, samples were shaken again using a vortex mixer and were centrifugated at 4 ºC until reaching maximum speed (14,600 rpm). Supernatant was separated from the pellet and were centrifuged at 14,600 rpm for 10 minutes in a new Eppendorf. The resultant supernatant was placed into a new Eppendorf and were kept on ice during 30 minutes in order to precipitate proteins. Afterwards, samples were centrifuged at maximum speed for 10 min. Supernatant was transferred to a new Eppendorf tube and evaporated until dryness under a gentle stream of nitrogen (N2) in a Sample Concentrator (Techne®, Staffordshire, UK). Subsequently, samples were kept at - 20 °C. Samples were reconstituted with 100 µL of ACN: H2O (90:10) + 1% FA and were centrifuged at maximum speed for 10 minutes. Finally, the solution was transferred into chromatographic vials for the subsequent LC-MS/MS analysis.

1.4.3. LC-MS/MS analysis

Metabolites were analysed using Ultra High Performance Liquid Chromatography couple to tandem mass spectrometry (UHPLC-MS/MS). The system consisted of an Acquity UPLC® H-class liquid chromatograph (Waters, Milford, MA, USA) coupled to a triple-quadrupole mass spectrometer (Xevo TQ-S micro). Chromatographic conditions were

based in previous reported studies based on the analysis of in zebrafish [7]. Separation was achieved using an Acquity UPLC BEH Amide (150 mm × 2.1 mm, 1.7 µm) and an Acquity UPLC BEH Amide pre-column (5 mm × 2.1 mm, 1.7 µm) (Waters, Milford, MA, USA). Mobile phase consisted of Milli-Q water and acetonitrile (H2O:ACN; 95:5) containing 100 mM ammonium formate (solvent A) and Milli-Q water and acetonitrile (H2O:ACN; 15:85) containing 30 mM ammonium formate (solvent B). Both solvents were adjusted to pH 3 with FA (SensionTM + PH3, HACH®, USA). Gradient elution program allowed the optimal separation of target metabolites starting at 100% B. Then, decreased to 80% B in 4 min and held for 1 min. In the following 2 min, solvent B was increased to 100%. Finally, initial conditions were re-equilibrated in 3 min, resulting in a total run time of 10 min. The flow rate was set at 250 µL min-1. Aliquots of 10 µL of standard and/or samples were injected at 10 ± 5°C.

Regarding MS conditions, cone gas flow was performed at 150 L h-1 and desolvation gas flow was set to 900 L h-1. Source temperature was set at 100 °C and desolvation temperature was 350 °C. Capillary voltage of 2.0 kV was applied. Metabolites were measured under positive electrospray ionization (ESI+), using N2 as desolvation and cone gas. The acquisition mode was selected reaction monitoring (SRM) mode using two transitions for each analyte. The first transition was used as the quantifier ion, whereas the second transition was used as qualifier ion. Experimental data were acquired and processed using MassLynx® v4.1 software package (Waters, USA).

1.4.4. Quality assurance

Calibration was performed over a concentration range from 0.005 to 2.5 ng µL-1. The ISM was used as extraction and analytical quality control. Instrumental detection limits (IDLs) were determined using the lowest concentrated standard 0.005 ng µL-1 that yielded a S/N ratio equal to 3. While method detection limits (MDLs) were calculated using samples spiked at 100 ng (quality controls, QCs). Intra-day precision was determined by four consecutive injections of 1 ng µL-1 standard solution, whereas inter-day precision was determined by measuring the same standard solution for three different days. Moreover, recovery studies were performed with four replicates, using QCs with the metabolite's standard mixture and the ISM. Moreover, matrix effect (ME) was assessed by comparing the peak area of each metabolite from QCs (A) with the peak area of the analyte from the standard solution used in calibration curve (C) according to the equation: ME (%)=(A-B)/C × 100, where B is the peak area of each analyte from non-spiked samples (controls). This parameter is an indicator of ionization suppression or enhancement of the analytes.

1.4.5. Quality parameters

Great correlation coefficients (r2) were obtained over 0.99 for all analytes in a range from 0.005 to 2.5 ng µL-1 in most cases, performing calibration curve with internal standard correction. Furthermore, IDLs were ranged from 0.40 pg (Chol) to 138.0 pg (NE), while MDLs varied from 0.4 (Chol) to 79.8 (5-HTP) pg Daphnia-1. Intra-day precision ranged from 0.4% to 5.5% and inter-day precision values were from 1.8% to 25.5%. Regarding matrix effect, compounds with values below 70% indicated signal suppression due to the matrix (Phe and Glut), whereas values above 130% suggested a signal enhancement (GABA). Table S2 summarizes the quality parameters obtained for each target metabolite.

2. Supplementary Results

Table S1. Analyzed monoamine neurotransmitters and related metabolites.

Pathway/Name	Abbrev.	KEGG number
**Cholinergic**		
Choline	Chol	C00114
Acetylcholine	ACh	C01996
**Serotonergic**		
L-Tryptophan	Trp	C00078
5-Hydroxy-L-tryptophan	5-HTP	C00643
Serotonin	5-HT	C00780
5-Hydroxyindoleacetic acid	5-HIAA	C05635
**Catecholaminergic**		
Phenylalanine	Phe	C00079
Tyrosine	Tyr	C00082
Tyramine	Tyra	C00483
Octopamine	Oct	
3,4-Dihydroxy-L-phenylalanine	LDOPA	C00355
Dopamine	DA	C03758
3-Methoxytyramine	3-MT	C05587
L-Noradrenaline	NE	C00547
**GABAergic**		
L-Glutamate	Glu	C00025
γ-Aminobutyric acid	GABA	C00334

Table S2. Quality parameters of monoamine neurotransmitters and related metabolites.

Compound	Linear range (ng μL^{-1})	Internal standard	F	R^2	IDL (pg)	Intra-day precision (RSD, %)	Inter-day precision (RSD, %)	Recovery ± RSD (%)	ME ± RSD (%)	MDL (pg *Daphnia*$^{-1}$)
Chol	0.005 – 2.5	Choline-^{13}C	0.42	0.9951	0.4	5.5	17.5	84±14	89±1	0.4
ACh		ACh-d^9	0.05	0.9939	1.6	1.6	12.8	98±4	74±11	1.9
Trp		Try-^{13}C	0.50	0.9979	2.6	3.6	1.8	78±18	77±19	6.6
5-HTP		LDOPA-d^3	2.41	0.9979	49.2	2.0	6.8	68±17	79±20	79.8
5-HT		DA-d_4	0.17	0.9987	26.4	0.4	4.1	60±8	115±17	19.3
5-HIAA		LDOPA-d^3	1.49	0.9962	107.0	4.1	25.5	82±14	97±22	3.3
Phe		Phe-^{13}C, ^{15}N	0.45	0.9962	3.3	4.7	10.5	136±14	64±2	3.2
Tyr		Tyr-$^{13}C_9$	1.46	0.9946	24.9	1.7	2.0	63±4	75±20	7.0
Tyra		3-MT-d_4	1.44	0.9968	1.1	5.2	8.8	110±5	79±4	23.8
Oct		ACh-d^9	0.06	0.9936	10.0	4.4	14.2	109±7	81±10	3.4
LDOPA		LDOPA-d^3	2.76	0.9982	5.4	4.4	13.0	71±6	74±15	20.5
DA		DA-d_4	0.14	0.9953	121.8	4.9	6.0	119±4	96±5	15.9
3-MT		3-MT-d_4	2.82	0.9947	0.7	5.4	12.7	100±5	79±7	4.1
NE		NE-d_6	1.59	0.9957	138.0	3.7	3.5	104±7	99±8	7.5
Glu		DA-d_4	0.59	0.9990	25.2	3.6	20.1	95±5	68±18	11.7
GABA		DA-d_4	0.24	0.9945	47.5	5.3	19.4	65±16	145±9	3.1

Table S3. Student's test results for measured MAO activity across three identical experiments and monoamine neurotransmitters across control and deprenyl treatments.

	df	t	P
MAO			
Exp1	3	7.2	0.005
Exp2	7	6.1	<0.001
Exp3	5	4.1	0.009
Metabolites			
Chol	8	-0.3	0.774
Acetylc	8	0.5	0.615
Trp	8	1.0	0.356
5HT	8	-3.0	0.017
5HIAA	8	0.8	0.473
Phe	8	0.4	0.692
Tyr	8	-0.6	0.59
Tyra	8	0.5	0.622
Oct	8	-0.3	0.803
LDOPA	8	-0.9	0.413
DA	8	-3.2	0.012
3MT	8	-2.7	0.027
NE	8	0.8	0.463
Glut	8	-0.6	0.547
GABA	8	-1.4	0.205

Table S4. Results for the non-significant affected monoamine neurotransmitters and metaboliles (Mean SE, N=5). Units are ng/mg protein except Ach, 5-HTP, Oct, NE that are pg/mg protein. C, control.

Metabolite	Treatment	N	Mean	SE
Chol	C	5	1433.4	114.7
	Deprenyl	5	1487	138.9
ACh*	C	5	6342.4	429.2
	Deprenyl	5	6077.3	269.7
Trp	C	5	485.7	108.9
	Deprenyl	5	377.7	16.1
5-HTP*				
5HIAA	C	5	70.8	6.7
	Deprenyl	5	61	11.2
Phe	C	5	694.5	117
	Deprenyl	5	642.8	46.2
Tyr	C	5	987.5	164.6
	Deprenyl	5	1092.8	89.8
Tyra	C	5	17.4	8.3
	deprenyl	5	13	2.3
Oct*	C	5	1282.9	140.9
	Deprenyl	5	1388.9	386
LDOPA	C	5	8.7	1.8
	Deprenyl	5	11.4	2.5
NE*	C	5	681.2	61.3
	Deprenyl	5	583.6	110.6
Glut	C	5	601.3	65.5
	Deprenyl	5	649.9	41
GABA	C	5	169.6	17.9
	Deprenyl	5	245	51.7

3. Supplementary References

1. Simão, F.C.P.F.C.P.; Martínez-Jerónimo, F.; Blasco, V.; Moreno, F.; Porta, J.M.J.M.J.M.; Pestana, J.L.T.J.L.T.; Soares, A.M.V.M.A.M.V.M.A.M.V.M.; Raldúa, D.; Barata, C. Using a new high-throughput video-tracking platform to assess behavioural changes in Daphnia magna exposed to neuro-active drugs. Sci. Total Environ. 2019, 662, 160–167, doi:10.1016/j.scitotenv.2019.01.187.
2. Barata, C.; Baird, D.J. Phenotypic plasticity and constancy of life-history traits in laboratory clones of Daphnia magna straus: Effects of neonatal length. Funct. Ecol. 1998, 12, 442–452, doi:10.1046/j.1365-2435.1998.00201.x.
3. Bedrossiantz, J.; Martínez-Jerónimo, F.; Bellot, M.; Raldua, D.; Gómez-Canela, C.; Barata, C. A high-throughput assay for screening environmental pollutants and drugs impairing predator avoidance in Daphnia magna. Sci. Total Environ. 2020, 740, doi:10.1016/j.scitotenv.2020.140045.
4. Holt, A.; Sharman, D.F.; Baker, G.B.; Palcic, M.M. A continuous spectrophotometric assay for monoamine oxidase and related enzymes in tissue homogenates. Anal. Biochem. 1997, 244, 384–392, doi:10.1006/abio.1996.9911.
5. Faria, M.; Prats, E.; Bellot, M.; Gomez-Canela, C.; Raldúa, D. Pharmacological modulation of serotonin levels in zebrafish larvae: Lessons for identifying environmental neurotoxicants targeting the serotonergic system. Toxics 2021, 9, doi:10.3390/toxics9060118.
6. Fuertes, I.; Barata, C. Characterization of neurotransmitters and related metabolites in Daphnia magna juveniles deficient in serotonin and exposed to neuroactive chemicals that affect its behavior: A targeted LC-MS/MS method. Chemosphere 2021, 263, 127814, doi:10.1016/j.chemosphere.2020.127814.
7. Mayol-Cabré, M.; Prats, E.; Raldúa, D.; Gómez-Canela, C. Characterization of monoaminergic neurochemicals in the different brain regions of adult zebrafish. Sci. Total Environ. 2020, 745, 141205, doi:10.1016/j.scitotenv.2020.141205.
